# A Fatal Case of Staphylococcus capitis Endocarditis in a Patient With Transcatheter Aortic Valve Replacement

**DOI:** 10.7759/cureus.35333

**Published:** 2023-02-22

**Authors:** Rojin Esmail, Curtis Ober, Chelsea Dunn, Damian Casadesus

**Affiliations:** 1 Internal Medicine, Ross University School of Medicine, St. Michael, BRB; 2 Internal Medicine, Jackson Memorial Hospital, Miami, USA

**Keywords:** transcatheter aortic valve replacement, aortic stenosis, aortic valve replacement in chronic kidney disease, staphylococcus capitis, end stage renal disease (esrd), tavr, endocarditis

## Abstract

Transcatheter aortic valve replacement (TAVR) has evolved to become a standard management modality for high-risk, moderate, and even low-risk patients with symptomatic aortic stenosis. Infective endocarditis (IE) after a TAVR is rare and difficult to diagnose. Typical sonographic characteristics observed with an echocardiogram in native valve endocarditis may not be present in TAVR-IE cases. Enterococcal species are identified to be the most frequent causative agents. Coagulase-negative staphylococci (CoNS) can infrequently lead to a fatal course of endocarditis in the TAVR population. There are only seven previously reported cases of *Staphylococcus capitis* (*S. capitis*) prosthetic valve endocarditis noted in the literature. Here we present a man in his 60s who presented to our facility for evaluation of fever and shortness of breath. He was subsequently diagnosed with *S. capitis* TAVR-IE. He was not considered a surgical candidate and was treated medically for IE with a fatal outcome.

## Introduction

Over the past decade, transcatheter aortic valve replacement (TAVR) has become the standard of care for high-risk and inoperable patients with symptomatic aortic stenosis. TAVR can also be considered alternative management in intermediate and low-risk patients [1]. Early or late onset of infective endocarditis (IE) after a TAVR is rare, difficult to diagnose, and often fatal. Given that this is relatively a new procedure, not much data has been reported on the best way to diagnose and manage this high-risk group [2]. Coagulase-negative staphylococci (CoNS) are known for their ability to form biofilms and adhere to the foreign indwelling and implanted medical devices [3]. However, unlike other CoNS, *Staphylococcus capitis* (*S. capitis*), which colonizes human skin and hair, has a lower ability to adhere to foreign medical devices, making it an uncommon and opportunistic cause of TAVR-IE.

## Case presentation

A male, in his 60s with a previous history of end-stage renal disease, early dementia, coronary artery bypass graft, and TAVR in 2014, presented to the emergency room with shortness of breath. The dyspnea began with exertion one week prior and progressed to dyspnea at rest. Vitals signs at admission showed a temperature of 37.2 °C, respiratory rate of 26 per minute, blood pressure of 80/60 mmHg, and heart rate of 110 beats per minute. On physical examination, the patient was noted to have jugular venous distention, a grade 2/6 systolic murmur at the right second intercostal space, and reduced but clear lung sounds on auscultation. Laboratory studies results were significant for a white cell count of 17,000 U/L and blood cultures that grew* S. capitis*.

**Figure 1:**
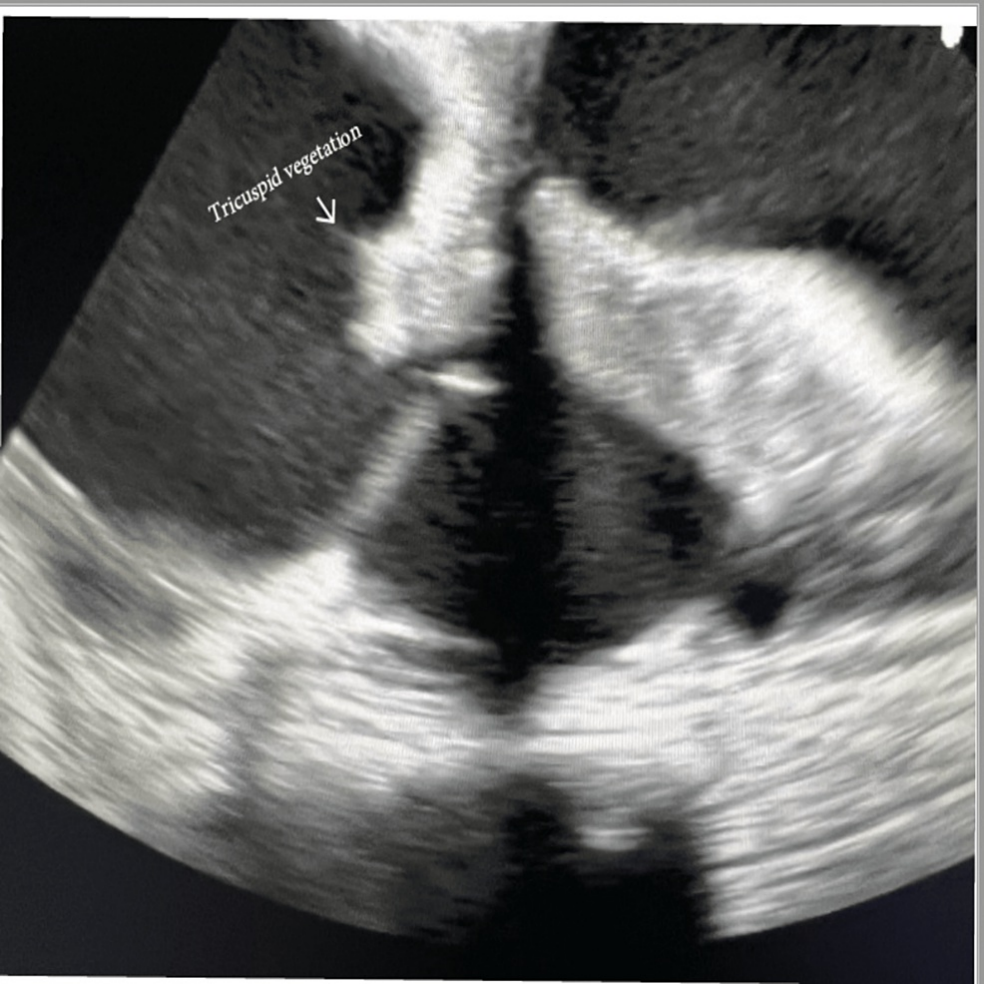
Tricuspid vegetation observed on transesophageal echocardiogram.

**Figure 2:**
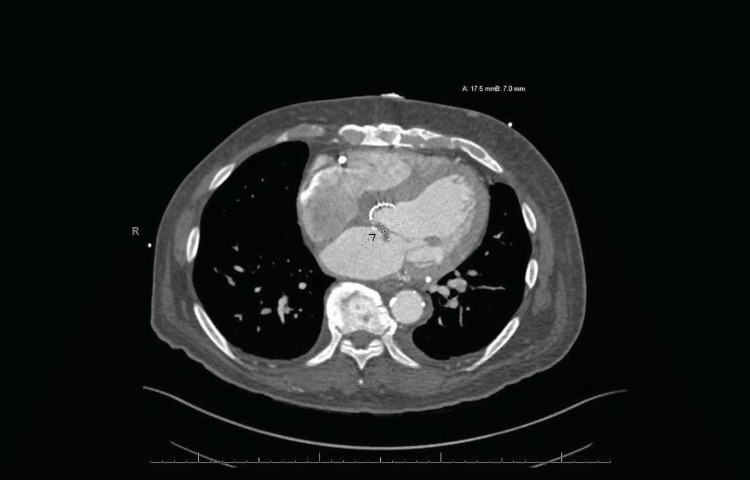
Mitral valve: Adherent to the left atrial surface of the anterior leaflet of the mitral valve, there is a 1.6 x 0.6 cm hypodense filling defect, that is highly suggestive of mitral valve vegetation. Also noted is a thickening of the tip of the anterior mitral valve leaflet measuring up to 5 mm in thickness. On cine images, the mitral valve seems to open adequately but there is incomplete coaptation of valve leaflets at systole consistent with mitral valve insufficiency.

**Figure 3:**
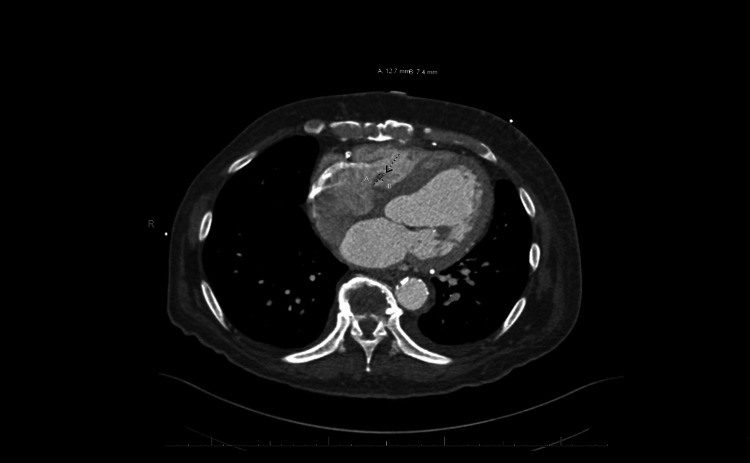
Tricuspid valve: Asymmetric thickening of the tricuspid valve septal leaflet by an adherent 1.3 x 0.7 cm nodular hypodense filling defect, that may represent tricuspid valve vegetation.

A computed tomography (CT) scan of the chest revealed multilobular pneumonia. A transthoracic echocardiogram (TTE) indicated an abnormal aortic valve gradient, thickened mitral valve leaflets with moderate to severe mitral valve, and moderate tricuspid regurgitation. Due to positive blood culture in presence of a TAVR, a transesophageal echocardiogram (TEE) was also obtained demonstrating a 1.9 cm x 0.6 cm vegetation of the tricuspid valve (Figure 1), severe stenosis of the prosthetic aortic valve, and severe mitral regurgitation with an ejection fraction (EF) of 40-45%. CT scan of the heart revealed vegetation in the left atrial surface of the anterior leaflet of the mitral valve (Figure 2), in addition to previously observed vegetation of the tricuspid valve septal leaflet (Figure 3), and severe stenosis and thickening of the aortic valve. The mitral valve vegetation that was observed on the CT scan was not previously observed on the TTE or TEE and even though no vegetation was observed on the TAVR, the patient was diagnosed with TAVR-IE in addition to mitral and tricuspid IE.

The patient wasn’t considered a surgical candidate due to a highly calcified aorta and early dementia, therefore he was treated medically with Cefazolin, Gentamicin, and Rifampin. On day 15 of hospitalization, he was taken to the cardiac catheterization department for structural heart evaluation and possible valve-in valve TAVR. At that time, the patient's fever and dyspnea were resolved. Catheterization revealed severe aortic stenosis, severe left anterior descending artery disease, and decreased cardiac output. Post-procedure, the patient developed cardiogenic and septic shock. He suffered cardiac arrest shortly after and was pronounced dead.

## Discussion

To the best of our knowledge, this is the first reported case of *S. capitis* as the cause of TAVR-IE. Incidence of TAVR-IE is rare (three to nine cases per 100,000 people) yet associated with high in-hospital mortality of 34% [4,5]. The most frequent causative agent of prosthetic valve endocarditis (PVE) in the TAVR population is Enterococcus [4]. CoNS is an infrequent cause of PVE in the TAVR population with the highest 30-day mortality at 31% compared to the other causative microorganism. Chronic hemodialysis is the most common risk factor present in IE populations and has the greatest association with CoNS [6].

*S. capitis* is an opportunistic species of CoNS that colonize the skin, hair, and nails of the human body. Its close evolutionary links to *Staphylococcus epidermidis* indicate that virulence and pathogenicity of *S. capitis* most likely include possession of genes that are important for the adhesion of bacteria to host, establishment, and maturation of biofilms on polymeric material and production of phenol-soluble modulins [3].

The diagnosis of IE after TAVR presents challenges that delay treatment and increase morbidity and mortality [2]. A study by Mangner et al. observed that 31.9% of patients with TAVR-IE had negative sonographic imaging [7]. In fact, our patient was subsequently diagnosed with TAVR-IE after his TEE only showed thickening and stenosis of the aortic valve without any vegetation. The TEE cannot rule out TAVR-IE because the higher density of the prosthetic valve prevents the passage of the ultrasound waves, and the shadow of the prosthetic valve further prevents visualization of the smaller vegetation and abscesses [7,8]. Additionally, TEE cannot provide a true assessment of paravalvular regurgitation. Regurgitation jets observed with TAVR-IE have different characteristics than native valves [7]. TAVR-IE may present obstructive patterns and leaflet thickening on the echocardiogram, which was observed in our patient [8].

*S. capitis* can be a fatal cause of TAVR-IE due to diagnostic and treatment challenges presented in this vulnerable population. Upon review of the literature, seven previously reported cases of surgical PVE were identified and they were all treated by surgical removal of the infected prosthesis (Table 1). A retrospective study by Tchana-Sato et al. concluded that early and appropriate antibiotic selection in addition to removal of the infected valve in the acute phase of the disease remains the best course of action for the management of the aggressive course of *S. capitis *PVE [9-12]. Yet, since high-risk patients after a TAVR are often not surgical candidates, treatment of *S. capitis* IE can particularly be challenging [2].

**Table 1:** Previously reported cases of surgical PVE PVE: prosthetic valve endocarditis

Age	Gender	Valve involved	Complications	Antibiotic selection	Surgery during the acute phase	Outcome	References
65	Female	Mitral PVE	NA	Imipenem and vancomycin	Yes	Survived	9
72	Male	Aortic PVE	Heart failure, Aortic root abscess	Vancomycin, gentamicin, and rifampicin	Yes	Died	10
48	Female	Aortic PVE	Heart failure, Embolic, Aortic root abscess	Vancomycin, gentamicin, and rifampicin	Yes	Died	10
79	Female	Aortic PVE	NA	Amukin, linezolid, teicoplanin, minocycline	Yes	Survived	11
79	Female	Aortic PVE	NA	Vancomycin and minocycline	Yes	Survived	11
76	Male	Aortic PVE	Aortic annular abscess	Teicoplanin, vancomycin, linezolid, levofloxacin	Yes	Survived	11
68	Female	Mitral PVE	Mitral annular abscess and Heart failure	Vancomycin, gentamycin, and levofloxacin	Yes	Survived	11

## Conclusions

A greater emphasis should be placed on prevention and prophylaxis measures of *S. capitis* TAVR-IE, due to its atypical presentation and treatment challenges. Currently, antibiotic prophylaxis for dental procedures after surgical or transcatheter placement of prosthetic valves is recommended. Transesophageal and transthoracic echocardiograms do not always show vegetation. The diagnosis of TAVR-IE should always be considered in a patient with bacteremia in the presence of TAVR, even with the images do not show the presence of the vegetation. Further research on implementing strict aseptic techniques and antibiotic prophylaxis protocol for patients at high risk of endocarditis such as our patient on chronic hemodialysis could be interesting to future research.
